# Relatively low tooth replacement rate in a sauropod dinosaur from the Early Cretaceous Ruyang Basin of central China

**DOI:** 10.7717/peerj.12361

**Published:** 2021-10-27

**Authors:** Huali Chang, Hai-Lu You, Li Xu, Waisum Ma, Diansong Gao, Songhai Jia, Mengli Xia, Jiming Zhang, Yu Li, Xirui Wang, Di Liu, Jie Li, Jianhua Zhang, Lili Yang, Xuefang Wei

**Affiliations:** 1 Henan Natural History Museum, Zhengzhou, China; 2 Key Laboratory of Vertebrate Evolution and Human Origins of Chinese Academy of Sciences, Institute of Vertebrate Paleontology and Paleoanthropology, Chinese Academy of Sciences, Beijing, China; 3 College of Earth and Planetary Sciences, University of Chinese Academy of Sciences, Beijing, China; 4 CAS Center for Excellence in Life and Paleoenvironment, Beijing, China; 5 School of Geography, Earth and Environmental Sciences, University of Birmingham, Birmingham, United Kingdom; 6 Institute of Geology, Chinese Academy of Geological Sciences, Beijing, China

**Keywords:** Sauropod, Tooth replacement rate, Early Cretaceous, Ruyang Basin, Henan

## Abstract

Tooth replacement rate is an important feature related to feeding mechanics and food choices for dinosaurs. However, only a few data points are available for sauropod dinosaurs, partially due to rarity of relevant fossil material. Four somphospondylan sauropod species have been recovered from the Lower Cretaceous Aptian–Albian Haoling Formation in the Ruyang Basin, Henan Province of central China, but no cranial material has been reported except for a single crown. Here we report the discovery of the rostral portion of a left dentary with replacement teeth in its first five alveoli. Comparative anatomical study shows the partial dentary can be assigned to a member of early diverging somphospondylans. The non-destructive tooth length-based approach to estimating tooth formation time and replacement rate is adopted here. The estimated tooth replacement rate is 76 days, faster than that of *Brachiosaurus* (83 days) and much lower than typical late diverging lithostrotian titanosaurians (20 days). Thus, this discovery adds an intermediate tooth replacement rate in the evolution of titanosauriform sauropods and supports the idea that evolution of tooth replacement rate is clade-specific. This discovery also provides more information to understand the Ruyang sauropod assemblage, which includes one of the most giant dinosaurs to have walked our Earth (*Ruyangosaurus giganteus*).

## Introduction

Tooth replacement rate is an important feature related to feeding mechanics and food choices for polyphyodonty animals, including dinosaurs. For example, *Camarasaurus* and *Diplodocus* have tooth replacement rates of about one tooth every 62 days *versus* 35 days, respectively, and this difference could help understand the coexistence of these gigantic herbivorous in the Upper Jurassic Morrison Formation land ecosystem (D’Emic et al., 2013). Tooth replacement rate is defined as the time required to replace one tooth in a given alveolus, and can be calculated by subtracting tooth formation times for successive teeth within one tooth family (D’Emic et al., 2013; Erickson, 1996). The non-destructive tooth length-based approach to estimating tooth formation time and replacement rate developed by D’Emic et al. (2013) has proven useful to estimate two different types of sauropod teeth, the broad-crowned and narrow-crowned, and this method is adopted here.

Within dinosaurs, tooth replacement rates have been shown to be clade-specific, with elevated rates in abelisaurid theropods, hadrosaurid ornithischians, and diplodocoid and late diverging titanosauriform sauropods (D’Emic et al., 2019). Among titanosauriforms, tooth replacement data are only available for three taxa: two for its earliest-diverging clade, the Brachiosauridae (92 and 83 days for *Giraffatitan* and *Brachiosaurus*, respectively), and one (20 days) for a Late Cretaceous titanosaur (D’Emic & Carrano, 2020; D’Emic et al., 2013; Kosch et al., 2014). Here, based on the discovery of one rostral portion of a left dentary and associated replacement teeth, a 76 days tooth replacement rate is estimated for a somphospondylan titanosauriform sauropod from the late Early Cretaceous of Ruyang Basin, Henan Province, central China. Therefore, this discovery provides more information to understand the evolution of tooth replacement rate in titanosauriform sauropods and enrich our knowledge on the Ruyang gigantic sauropod assemblage.

## Materials & Methods

**Figure 1:**
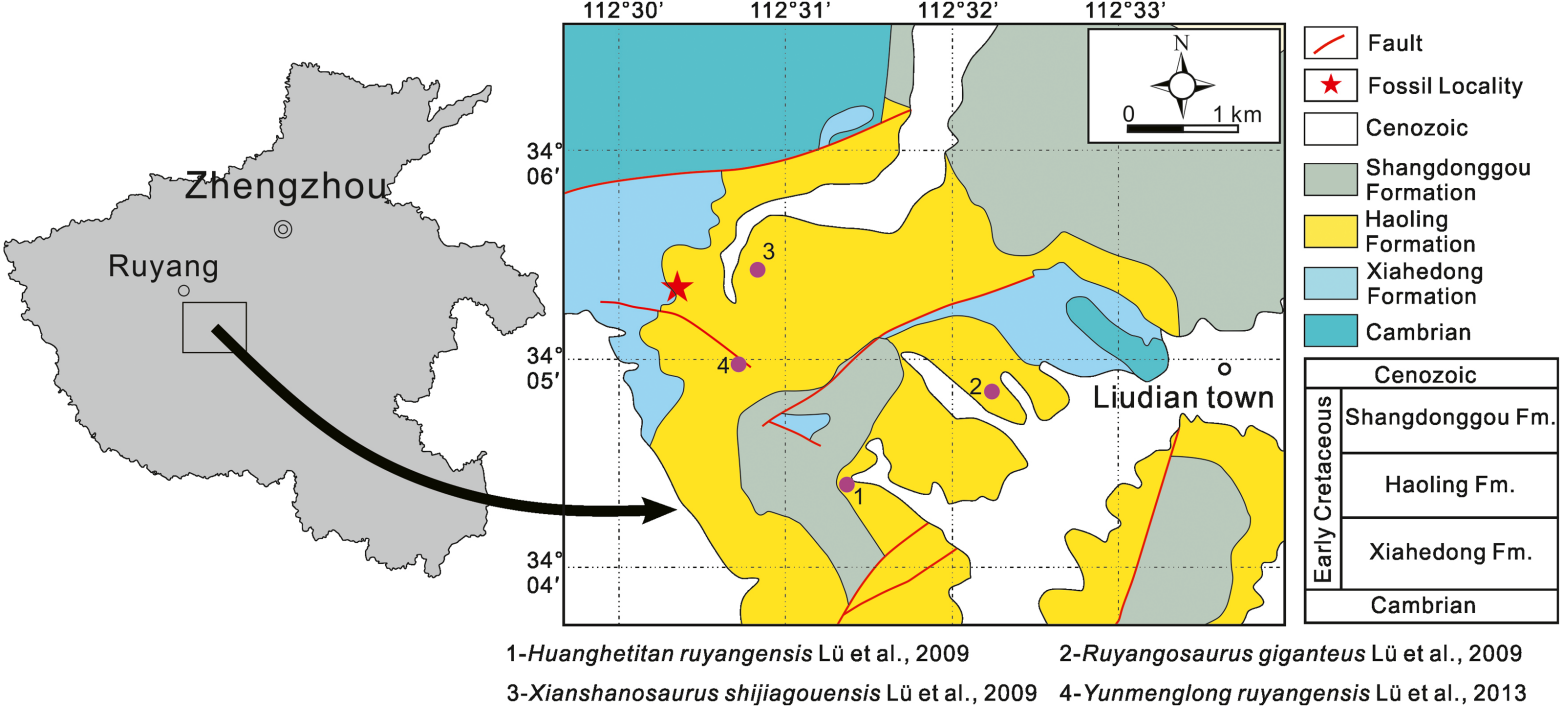
Geographical and geological maps showing the location of 41HIII-0016 and four sauropod species in the Lower Cretaceous Haoling Formation of Ruyang Basin, Henan Province, central China.

Henan Natural History Museum 41HIII-0016 (Field number: KLR08-1): rostral portion of a left dentary with replacement teeth in the first five alveoli, discovered from Shaping Village, Liudian Town, Ruyang County, Henan Province. Lower Cretaceous Aptian–Albian, Haoling Formation (Xu et al., 2010) (Fig. 1).

The partial dentary was computed tomography (CT) scanned at the Institute of Geology, Chinese Academy of Geological Sciences, Beijing, China, using a Nikon XTH225ST scanner. The specimen was scanned at 170 kV and 115 uA. The final data set contains 1,743 image slices (1,743 × 2,007 pixels). The CT data was imported into digital visualization software Avizo (version 9.1) for image processing. Dental elements were segmented using segmentation editor in Avizo, and 3D surface models were then imported to Blender (version 2.79) for optimization.

The raw CT scan and reconstructed 3D models of 41HIII-0016 are available on MorphoSource at the following DOIs: 10.17602/M2/M369117, 10.17602/M2/M365661, 10.17602/M2/M365525.

The non-destructive tooth length-based approach to estimating tooth formation time and replacement rate developed by D’Emic et al. (2013) is adopted here. The incremental line thickness is assumed to be similar to those of *Camarasaurus* and *Diplodocus*. Tooth formation time (days) is calculated according to the algorithm derived for *Camarasaurus*, y =−0.0078x^2^ + 2.6771x + 109.44, where x = tooth length, and tooth replacement rate is calculated by subtracting tooth formation times for successive teeth (D’Emic et al., 2013; Erickson, 1996).

## Results

### Description

**Figure 2:**
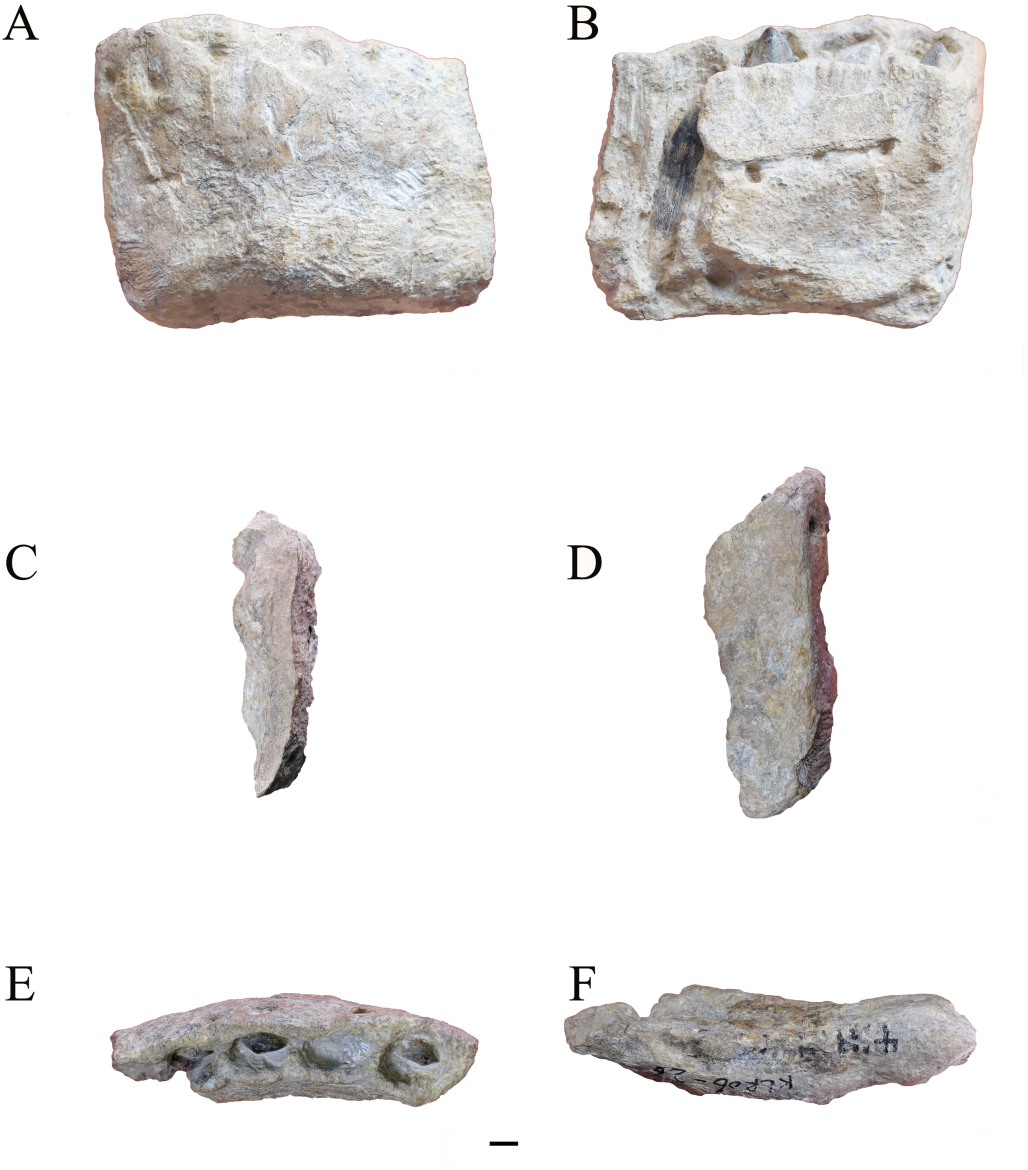
Photograph of 41HIII-0016, the rostral portion of a left dentary. (A) Lateral. (B) Medial. (C) Caudal. (D) Rostral. (E) Dorsal. (F) Ventral views. Note fragment tooth in alveolus 5 has lost after the CT scanning.

**Figure 3:**
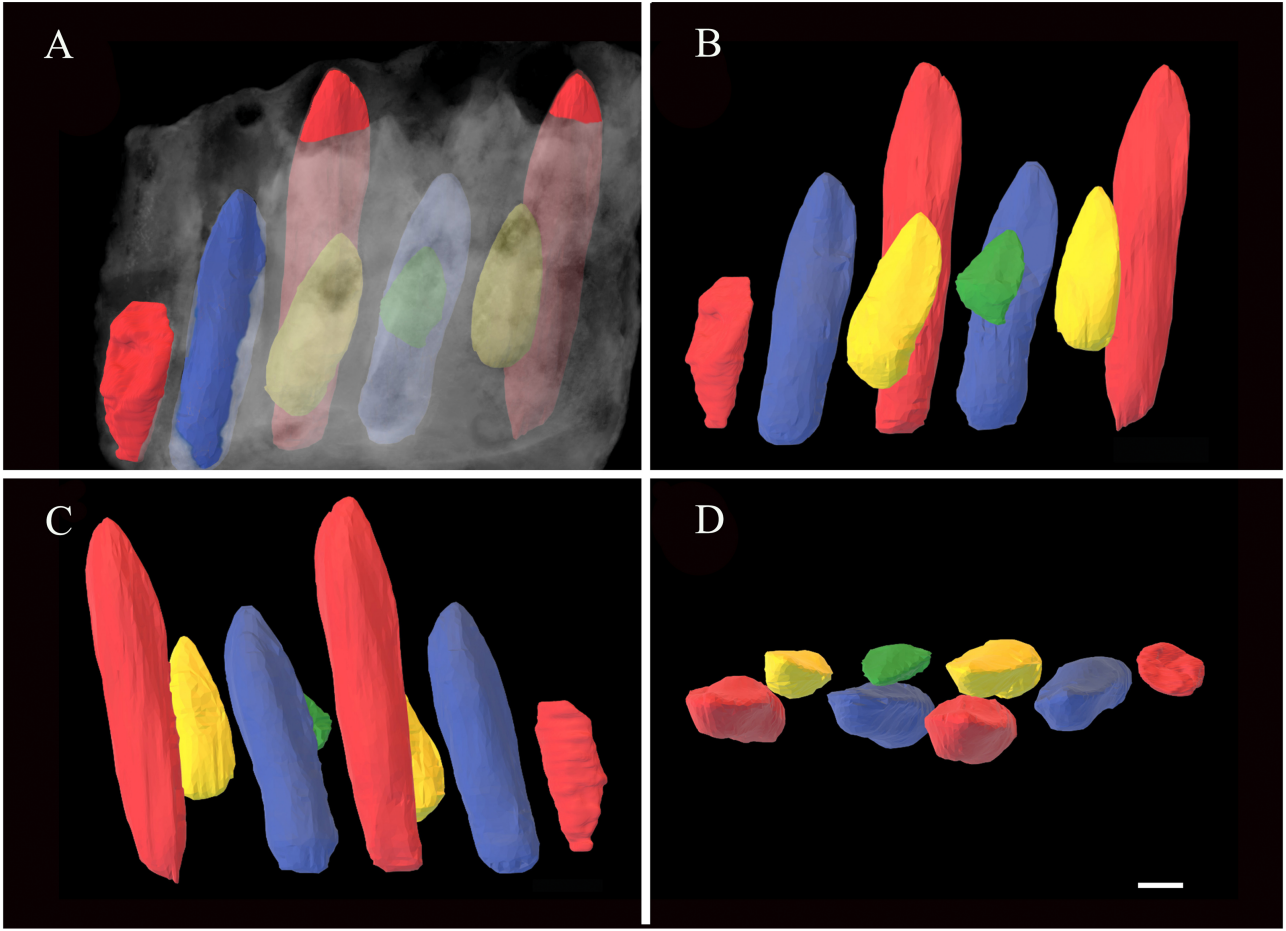
Reconstruction 3D mode of the replacement teeth in 41HIII-0016. (A) Medial with bones in shadow. (B) Medial. (C) Lateral. (D) Dorsal views. Scale bar equals 1 cm.

The 123 mm long partial left dentary includes the first five alveoli and the symphysis (Figs. 2 and 3). The preserved portion slightly increases in height rostrally, from 84 to 95 to 101 mm across the fifth, third and first alveoli, respectively, and its thickness does not change generally, approximately 2.6 times taller than broad. In dorsal view, it arches outwardly slightly, and judging from the orientation of the symphysis, it would direct around 45 degrees caudolaterally. The vertically oriented symphyseal surface is oblong, 101 mm height, and narrows slightly ventrally, from 31 mm at its dorsal edge to 21 mm at the ventral end. The symphysis protrudes slightly more ventrally than the remainder of the dentary, and bears a rostrodorsal-caudoventrally oriented groove, which can be see clearly in medial view.

In lateral view, a row of six large foramina is roughly aligned at the level of the ventral margin of the lateral plate, two associated with the first alveolus and the other four each with the following four alveoli, respectively. The lateral plate is about 15 mm high above the level of the dorsal edge of the paradental plates on the medial surface. Caudal to the first alveolus, a shallow horizontal depression extends at the level just ventral to the md-height. On the medial surface three foramina can be seen along the paradental groove, which is positioned higher than the mid-height of the medial surface and parallel to the dorsal edge. This groove does not extend onto the first alveolus. Below the paradental groove, the Meckelian groove is evident on the ventral end, starting from the second alveolus and increasing in height caudally.

No fully erupted teeth are preserved. Replacement teeth can be seen medially on alveoli 1, 3, 4 and 5, respectively. In dorsal view, the boundary of alveolus 1 is as long as wide, but in subsequent alveoli, their widths decrease slightly. CT scanning reveals the typical alternating pattern of tooth replacement, and two replacement teeth can be seen in alveoli 1, 2, and 3, respectively. Based on their lengths, four developmental stages can be cataloged, and stages 1 and 3 can been seen in alveoli 1 and 3, while stages 2 and 4 can been seen in alveoli 2 and 4 (Table 1).

**Table 1:** Measurements (in mm), tooth formation times (days) and replacement rate (days) for 41HIII-0016 (KLR08-1).

Alv	Sta	L	W	L/W	TFT	TRR
1	1	82.9	16.3	5.08	277.8	
1	3	37.6	15.3	2.46	199.1	
						78.7
2	2	60.4	16.2	3.73	242.5	
2	4	20.7	12.4	1.67	161.5	
						81.0
3	1	81.1	16.0	5.07	275.3	
3	3	41.0	14.8	2.77	206.1	
						69.2
4	2	60.8	16.3	3.73	243.4	
Average tooth replacement rate						76.3

**Notes.**

Abbreviations Alv alveolus Sta developmental stages based on tooth lengths L tooth length TFT tooth formation times TRR tooth replacement W tooth maximal width

Tooth formation time (days) is calculated according to the algorithm derived for Camarasaurus. (y =  − 0.0078x^2^ + 2.6771x + 109.44; x = tooth length).

Based on the longest replacement teeth in alveoli 1 and 3, the crown is not expanded mesiodistally, and about the same width at its junction to the root. The crown keeps its width for its basal half and tapers apically. The cross section around the mid-height crown is D-shaped, with a strongly convex labial side and roughly flat lingual side. Apicobasally extended ridges can be seen along the midlines on both the labial and lingual surfaces, and the lingual one is more developed than the labial one.

## Discussion

### Phylogenetic position

The tooth shape, the broadly arched, more U - than V -shaped rostral portion of the mandible in dorsal view, and the large size show this rostral portion of the dentary belongs to a sauropod dinosaur (Rauhut, 2003; Upchurch, Barrett & Dodson, 2004; Barrett & Upchurch, 2007). Among sauropods, the lack of “chin” at the rostroventral corner of the dentary, non-transversely oriented rostral portion of the dentary, and less than three replacement teeth per alveolus indicate it does not belong to Diplodocoidea (Upchurch, Barrett & Dodson, 2004; Whitlock, 2011; D’Emic & Carrano, 2020).

The mesiodistally narrow and D-shaped cross section of the crown, lack of denticles along the mesial and distal margins, and closeup of the rostral end of the Meckelian groove to the symphysis show the specimen probably belongs to a member of titanosauriforms (Upchurch, Barrett & Dodson, 2004; D’Emic & Carrano, 2020). Titanosauriformes includes Brachiosauridae and Somphospondyli, and the latter includes some early diverging taxa and Titanosauria, with Lithostrotia as the well-known latest diverging clade (Carballido et al., 2017; Mannion et al., 2019). The lack of the typical pencil-like narrow teeth with cylindrical crown cross section indicate the Ruyang dentary probably belongs to an early diverging non-lithostrotian titanosauriform.

The lower jaw and dentary teeth of *Brachiosaurus altithorax* (USNM 5730) were described (D’Emic & Carrano, 2020). The height and width of its right symphysis is 109 mm and 38 mm, respectively, similar to that of the Ruyang dentary (101 mm and 31 mm). Their general morphology and tooth shape are similar, but measurements of the dentary teeth show interesting differences. Among the comparable teeth in the first three alveoli, the *Brachiosaurus* teeth are more robust than the corresponding ones in the Ruyang specimen. For example, the length/width of the first stage in alveoli 1, 2, and 3 of *Brachiosaurus* are 64/16, 39+/21, and 60/20, while those of the Ruyang specimen are 82.9/16.3, 60.4/16.2, and 81.1/16.0 (mm). Therefore, the Ruyang teeth are more slender and closer morphologically to the teeth of late diverging titanosauriforms than the *Brachiosaurus*-clade. Moreover, the tooth replacement rates of the Ruyang teeth (see below) are faster than that of the *Brachiosaurus* but slower than the late diverging titanosauriforms, such as the Rio Negro specimen (D’Emic et al., 2013).

*Euhelopus* is a relatively small sauropod, and the height of its symphysis is about half that of the Ruyang symphysis (Poropat & Kear, 2013; Wilson & Upchurch, 2009; Wiman, 1929). The symphyseal surface is tilted approximately 10 degrees from vertical in *Euhelopus*, while it is about vertically oriented in the Ruyang specimen. No replacement dentary teeth are known in *Euhelopus*, and their erupted teeth contact each other slightly. Although no erupted teeth are preserved in the Ruyang dentary, they would have not contacted to each other as suggested by the narrow replacement teeth. The *Euhelopus* crown expands slightly mesiodistally immediately adjacent to the root then tapering apically, while the Ruyang crown is almost parallel-sided around the crown-root junction in labial view. The Ruyang crown also lacks the rounded boss-like structure on the lingual part of each mesial and distal margin close to the base of the crown, a distinctive feature of *Euhelopus*.

*Liaoningotitan sinensis* from the Early Cretaceous Yixian Formation of Liaoning Province is represented by a well-preserved skeleton including low jaw (Zhou et al., 2018). Based on its preliminary description, the downturned rostral portion of the dentary strongly curves medially, forming an almost vertical angle to the rest of the dentary in dorsal view, and the symphyseal surface is not vertically oriented. The dentary teeth do not contact to each other but with a large spacing about one tooth wide, and the cross section of the narrowed crown are elliptical. Phylogenetic analysis posits *Liaoningotitan* between *Euhelopus* and Lithostrotia (Zhou et al., 2018). The Ruyang taxon seems to be in a position latter diverging than *Euhelopus* but earlier diverging than *Liaoningotitan* based on its tooth morphology and not strongly curved rostral portion of dentary in dorsal view.

### Tooth formation times and tooth replacement rates

Tooth lengths have been used to estimate their formation times with two algorithms derived from *Camarasaurus* and *Diplodocus*, respectively (D’Emic et al., 2013). Because the Ruyang taxon does not belong to a diplodocoid, the algorithm for *Camarasaurus* is used here.

**Figure 4:**
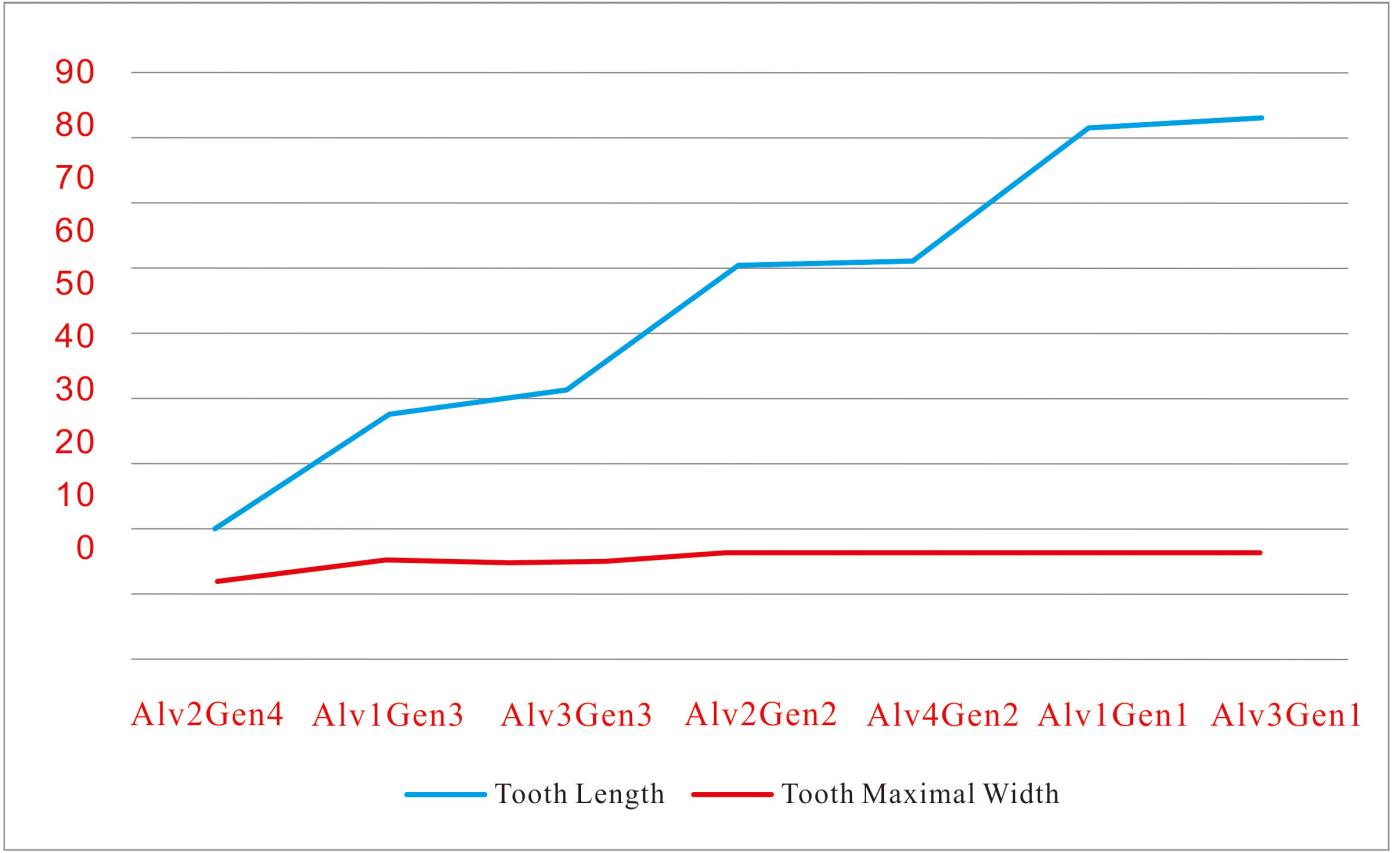
Tooth length increasing a step by ∼20 mm in successive four developmental stages while their width keeping roughly the same in 41HIII-0016. (in mm).

Interestingly, lengths increase by a step of around 20 mm, from an average of 20.7 to 39.3 to 60.6 to 82.0 mm from stages 1 to 4 in 41HIII-0016 (Fig. 4). Accordingly, the tooth formation times increase from 161.5 to 202.6 to 243.0 to 276.6 days (Table 1). This implies a speedup of daily tooth length increase with growth. Another aspect is that the widths of the teeth increase slightly with growth, and in the last two stages, their widths are about the same (around 16.2 mm), although their lengths increased 21.4 mm.

The average tooth replacement rate of the Ruyang taxon is 76 days. This is much slower than that of the unnamed late diverging titanosauriform (20 days) and *Diplodocus* (34 days), slower than that of *Camarasaurus* (62 days), faster than that of *Brachiosaurus* (83 days) and *Giraffatitan* (92 days), and much faster than that of *Mamenchisaurus* (98 days) (D’Emic & Carrano, 2020; D’Emic et al., 2013). This is in consistent with our suggestion that the Ruyang taxon is mostly likely to be a member of early diverging Somphospondyli, but not a member of brachiosaurid.

### The late Early Cretaceous Ruyang sauropod assemblage

Four sauropod species, *Huanghetitan ruyangensis* (Lü et al., 2007), *Ruyangosaurus giganteus* Lü et al., 2009a, Lü et al., 2009b *Xianshanosaurus shijiagouensis* Lü et al., 2009a, Lü et al., 2009b, and *Yunmenglong ruyangensis* (Lü et al., 2013), have been recovered from the Lower Cretaceous Aptian-Albian Haoling Formation in the Ruyang Basin, Henan Province of central China. They include some of the most giant sauropods known worldwide so far. For examples, the width of the dorsosacral centrum of *Ruyangosaurus giganteus* reaches 68 cm, a single rib of the holotype of *Huanghetitan ruyangensis* reaches 2.93 meter long, and the femoral length of *Yunmenglong ruyangensis* is 192 cm. *Xianshanosaurus shijiagouensis* is a medium-sized sauropod, with a femoral length of 126 cm. All have been considered as early diverging somphospondylans (Lü et al., 2014).

Except for a partial tooth crown found in the same quarry of *Xianshanosaurus*, no other cranial and dental material has been reported for the Ruyang giant sauropod assemblage (Lü et al., 2009a, Lü et al., 2009b). The worn crown of *Xianshanosaurus* does show some similarity with 41HIII0016 described here. For example, both are narrow and have a lingual ridge. Phylogenetic analysis has recovered *Xianshanosaurus* as a relatively late diverging titanosauriform, close to Titanosauria (Mannion et al., 2019). If 41HIII-0016 has a close relationship with *Xianshanosaurus*, their features of dentary and dentition, such as narrowed crown and relative faster tooth replacement rate than that of *Brachiosaurus*, would represent an intermediate stage in the evolution of titanosauriforms. This partial dentary could represent the latest diverging sauropod among the Ruyang sauropod assemblage.

## Conclusions

This is the first discovery of cranial material with teeth for the late Early Cretaceous Ruyang giant sauropod assemblage. Comparative anatomical study suggests the partial dentary as a member of early diverging titanosauriforms. CT scan-based 3D model of its replacement teeth allowed an estimation of 76 days for its replacement rate, which is intermediate between those of Brachiosauridae and late diverging titanosauriforms; therefore, tooth replacement rate increases in the evolution of titanosauriform sauropods.

## Additional Information and Declarations

### Data Availability

The following information was supplied regarding data availability: The raw CT scan and reconstructed 3D models of 41HIII-0016 are available at MorphoSource: - 10.17602/M2/M369117, - 10.17602/M2/M365661, - 10.17602/M2/M365525.

## References

[ref-1] Barrett P, Upchurch P (2007). The evolution of feeding mechanisms in early sauropodomorph dinosaurs. Special Papers in Palaeontology.

[ref-2] Carballido JL, Pol D, Otero A, Cerda IA, Salgado L, Garrido AC, Ramezani J, Cúneo NR, Krause JM (2017). A new giant titanosaur sheds light on body mass evolution among sauropod dinosaurs. Proceedings of the Royal Society B: Biological Sciences.

[ref-3] D’Emic MD, Carrano MT (2020). Redescription of Brachiosaurid Sauropod Dinosaur Material from the Upper Jurassic Morrison Formation, Colorado, USA. The Anatomical Record.

[ref-4] D’Emic MD, O’Connor PM, Pascucci TR, Gavras JN, Mardakhayava E, Lund EK (2019). Evolution of high tooth replacement rates in theropod dinosaurs. PLOS ONE.

[ref-5] D’Emic MD, Whitlock JA, Smith KM, Fisher DC, Wilson JA (2013). Evolution of high tooth replacement rates in Sauropod Dinosaurs. PLOS ONE.

[ref-6] Erickson GM (1996). Incremental lines of von Ebner in dinosaurs and the assessment of tooth replacement rates using growth line counts. Proceedings of the National Academy of Sciences of the United States of America.

[ref-7] Kosch JCD, Schwarz-Wings D, Frisch G, Issever AS (2014). Tooth replacement and dentition in *Giraffatitan brancai*. Society of Vertebrate Paleontology Annual Meeting (Journal of Vertebrate Paleontology, Supplement).

[ref-8] Lü J-C, Pu H-Y, Xu L, Jia S-H, Zhang J-M, Shen C-Z (2014). Osteology of the giant sauropod dinosaur *Ruyangosaurus giganteus* Lü others,2009.

[ref-9] Lü J-C, Xu L, Jia S-H, Zhang X-L, Zhang J-M, Yang L-L, You H-L, Ji Q (2009a). A new gigantic sauropod dinosaur from the Cretaceous of Ruyang, Henan, China. Geological Bulletin of China.

[ref-10] Lü J-C, Xu L, Jiang X-J, Jia S-H, Li M, Yuan C-X, Zhang X-L, Ji Q (2009b). A preliminary report on the new dinosaurian fauna from the Cretaceous of the Ruyang Basin, Henan Province of central China. Journal of Paleontological Society of Korea.

[ref-11] Lü J-C, Xu L, Pu H-Y, Zhang X-L, Zhang Y-Y, Jia S-H, Chang H-L, Zhang J-M, Wei X-F (2013). A new sauropod dinosaur (Dinosauria, Sauropoda) from the late Early Cretaceous of the Ruyang Basin (central China). Cretaceous Research.

[ref-12] Lü J-C, Xu L, Zhang X-L, Hu W-Y, Wu Y-H, Jia S-H, Ji Q (2007). A new gigantic sauropod dinosaur with the deepest known body cavity from the Cretaceous of Asia. Acta Geologica Sinica (English Edition).

[ref-13] Mannion PD, Upchurch P, Jin X-S, Zheng W-J (2019). New information on the Cretaceous sauropod dinosaurs of Zhejiang Province, China: impact on Laurasian titanosauriform phylogeny and biogeography. Royal Society Open Science.

[ref-14] Poropat SF, Kear BP (2013). Photographic atlas and three-dimensional reconstruction of the holotype Skull of *Euhelopus zdanskyi* with description of additional cranial elements. PLOS ONE.

[ref-15] Rauhut O (2003). A dentary of *Patagosaurus* (Sauropoda) from the Middle Jurassic of Patagonia. Ameghiniana.

[ref-16] Upchurch P, Barrett PM, Dodson P, Weishampel DB, Dodson P, Osmólska H (2004). Sauropoda. The Dinosauria.

[ref-17] Whitlock JA (2011). Inferences of Diplodocoid (Sauropoda: Dinosauria) feeding behavior from snout shape and microwear analyses. PLOS ONE.

[ref-18] Wilson JA, Upchurch P (2009). Redescription and reassessment of the phylogenetic affinities of *Euhelopus zdanskyi* (Dinosauria: Sauropoda) from the Early Cretaceous of China. Journal of Systematic Palaeontology.

[ref-19] Wiman C (1929). Die Kriede-dinosaurier aus Shantung. Palaeontologia Sinica Ser. C.

[ref-20] Xu L, Zhang X-L, Lü J-C, Jia S-H, Pang Z-C, Qin S, Zhu H-W, Zeng G-Y (2010). The Ruyang gigantic sauropod dinosaurian fauna from Henan Province and discussion on geological time of the fossi-bearing strata. Geological Review.

[ref-21] Zhou C-F, Wu W-H, Sekiya T, Dong Z-M (2018). A new titanosauriformes dinosaur from Jehol Biota of western Liaoning, China. Global Geology.

